# Diagnostic Accuracy of Transvaginal Ultrasound for Endometriosis Does Not Differ Between Women Undergoing Their First Versus Any Subsequent Surgical Treatment

**DOI:** 10.1111/ajo.70171

**Published:** 2026-08-17

**Authors:** Anais Alonso, Cecilia Ng, Rebecca Deans, Grant W. Montgomery, Gita D. Mishra, Erin Nesbitt‐Hawes, Jason A. Abbott

**Affiliations:** ^1^ Gynaecological Research and Clinical Evaluation (GRACE) Unit, Royal Hospital for Women Sydney New South Wales Australia; ^2^ School of Clinical Medicine, Discipline of Women's Health University of New South Wales Sydney New South Wales Australia; ^3^ National Endometriosis Clinical and Scientific Trials (NECST) Network, University of New South Wales Sydney New South Wales Australia; ^4^ Ainsworth Endometriosis Research Institute (AERI), University of New South Wales Sydney, New South Wales Australia; ^5^ Institute of Molecular Bioscience, University of Queensland St Lucia Queensland Australia; ^6^ School of Public Health University of Queensland Herston Queensland Australia

**Keywords:** accuracy, diagnosis, disease recurrence, endometriosis, transvaginal ultrasound

## Abstract

**Background:**

Australian endometriosis guidelines recommend transvaginal ultrasound (TVUS) as the first‐line investigation for endometriosis. Where endometriosis has been surgically treated, there is a risk of recurrent disease and repeat surgery. It is unknown if the diagnostic accuracy of ultrasound for recurrent disease is equivalent to the surgery‐naïve.

**Aims:**

To compare the accuracy of TVUS to diagnose endometriosis in women undergoing a first versus any subsequent laparoscopy.

**Materials and Methods:**

Retrospective analysis of the National Endometriosis Clinical and Scientific Trials registry included women aged 18–50 years who had undergone TVUS prior to gynaecological laparoscopy. Diagnostic accuracy of TVUS was compared between women with and without a history of surgical endometriosis treatment, with laparoscopy and histopathology as the reference standards. Subgroup analysis was undertaken by disease phenotype.

**Results:**

There were 470 ultrasound–laparoscopy dyads included for analysis (first laparoscopy *n* = 245 vs. subsequent laparoscopy *n* = 225), with histopathology available for 443 cases (first laparoscopy *n* = 234 vs. subsequent laparoscopy *n* = 209). Diagnostic accuracy did not differ between the two groups when compared to either laparoscopy (first laparoscopy: AUC 0.58 [95% CI 0.31–0.85], sensitivity 40.7% [34.7%–47.0%], specificity 75.0% [29.0%–96.0%] vs. subsequent laparoscopy: AUC 0.60 [0.46–0.74], sensitivity 46.2% [39.6%–52.9%], specificity 73.3% [47.5%–89.3%]) or histopathology (first laparoscopy: AUC 0.54 [0.31–0.77], sensitivity 40.8% [34.6%–47.3%], specificity 66.7% [29.6%–90.4%] vs. subsequent laparoscopy: AUC 0.56 [0.45–0.67], sensitivity 47.5% [40.4%–54.8%], specificity 64.3% [45.7%–79.3%]).

**Conclusions:**

Diagnostic accuracy did not differ based on previous endometriosis surgery. Community‐based TVUS performs considerably more poorly than research suggests, missing 55.5% of endometriosis overall and 63.2% of superficial disease, reinforcing that a negative ultrasound does not exclude endometriosis.

## 1 Introduction

Laparoscopy is identified as the gold standard diagnostic technique for endometriosis given the requirement for histological findings of endometrial glands and stroma. This may lead to an overreliance on invasive surgical intervention, contributing to the diagnostic delay of 6–8 years in Australia [1, 2] and an underreporting of the true prevalence of disease. RANZCOG's 2025 Australian endometriosis guidelines [3] recommend that patients be managed empirically using a clinical diagnosis, although formalising a diagnosis may bring substantial relief to patients by validating their symptoms, removing fear of a more sinister diagnosis and providing pathways for medical and social support [4].

Previous studies report that in the hands of a highly skilled operator, transvaginal ultrasound (TVUS) is highly accurate in diagnosing endometriosis [5]. RANZCOG's Australian endometriosis guidelines recommend TVUS as the first‐line investigation, as it is less invasive than surgery and empiric medical treatment can commence without a histological diagnosis [3].

While laparoscopy significantly reduces long‐term pain associated with endometriosis, symptom recurrence is common, and 36% of women undergo repeat surgery within 5 years of initial laparoscopy [6]. It is recognised that pain recurrence is not predictive of disease presence, and the ability to identify disease recurrence non‐invasively would minimise unnecessary surgical intervention and risk, with reduced costs to the patient and healthcare system.

Currently, it is unknown if the diagnostic accuracy of TVUS for recurrent disease is equivalent to that of those undergoing their first laparoscopy, as no study to date has investigated this. The aim of this study is to compare the accuracy of TVUS in diagnosing endometriosis between women undergoing their first laparoscopy and those who have previously undergone surgical treatment for endometriosis using Australia's national endometriosis registry data.

## 2 Materials and Methods

Ethics approval for this study was granted by the Monash Health Human Research Ethics Committee A (reference no: RES‐20‐0000‐258A), and written electronic informed consent was obtained from all participants. This study was undertaken in adherence with the Standards for Reporting Diagnostic Accuracy [7]. This was a retrospective analysis of the National Endometriosis Clinical and Scientific Trials (NECST) registry, the protocol for which has been published elsewhere [8]. In summary, nationwide recruitment of women and those assumed female at birth with a diagnosis of endometriosis or adenomyosis, or experiencing endometriosis‐related symptoms was conducted by on‐site researchers and clinicians, patient advocate groups and social media. Contained within the registry are demographic information, medical history, investigations, current and past management, patient‐reported outcome measures and quality of life scores.

Women were eligible for inclusion in this study if they had enrolled in the NECST registry between December 2020 (registry inception) and September 2024, were aged 18–50 years at the time of laparoscopy and had undergone a transvaginal pelvic ultrasound prior to gynaecological laparoscopy (conventional or robotic‐assisted), with both reports available in the NECST registry. Women were excluded if they underwent only a transabdominal ultrasound, were pregnant at the time of ultrasound or laparoscopy or underwent ultrasound more than 10 years prior to laparoscopy. Histopathology reports from included laparoscopies were also collected, but women were not excluded if this was not available.

Participants were allocated to the first laparoscopy group if they had never undergone surgical treatment for endometriosis, as indicated by the patient at the time of enrolment and by the operation reports available in the NECST registry. This included women with a previous diagnostic laparoscopy with no endometriosis found or no surgical treatment performed. Those who had previously undergone surgical treatment for endometriosis (including incomplete treatment) were allocated to the subsequent laparoscopy group. Any participant who did not indicate their endometriosis surgery history and had no previous laparoscopy reports in the registry was excluded. Where multiple ultrasound–laparoscopy dyads were available for a single patient, each was analysed as a unique case. Where women had undergone multiple TVUS prior to any one laparoscopy, the most recent ultrasound was analysed. Surgeons were not masked to TVUS findings. A diagnosis of endometriosis or other pathology by any of the index or reference standards was based on the sonologist, surgeon or histopathologist's report. If any report was indeterminate or uninterpretable, the case was excluded.

Based on a reported prevalence of endometriosis of 14% [9] and a sensitivity and specificity of 79% and 91%, respectively, for TVUS [10], 455 participants would be required to power the study to 95% with 10% margin of error [11]. The data were analysed using International Business Machines Corporation (IBM) Statistical Product and Service Solutions Statistics for Windows Version 26 (IBM, New York, U.S.) and XLSTAT (Data Analysis and Statistical Solution for Microsoft Excel, Addinsoft, Paris, France). A generalised linear mixed model was performed to determine independence of the two study groups. Data were tested for normality using the Kolmogorov–Smirnov method. Categorical data were assessed using Fisher's exact and chi‐square tests, while numerical data were analysed using the independent‐samples *t*‐test and Mann–Whitney *U* test. The index test was TVUS, with laparoscopy and histopathology being the reference standards. Diagnostic accuracy was determined using accuracy, sensitivity, specificity, positive predictive value (PPV), negative predictive value (NPV), positive likelihood ratio (LR+), negative likelihood ratio (LR−) and area under the receiver operating characteristic curve (AUC), with corresponding 95% confidence intervals (CI). Little's Missing Completely at Random test was performed for missing histopathology results. Best‐case/worst‐case scenario analysis was subsequently undertaken; in the best‐case scenario, missing histopathology was assumed to be concordant with the ultrasound report regarding the presence of endometriosis; in the worst‐case scenario, missing histopathology was assumed to be discordant. Subgroup analysis was performed by phenotype of endometriosis (superficial, deep and ovarian endometrioma) – women were excluded from subgroup analysis if the endometriosis phenotype was not discernible from the operation report. As classification of depth of disease was not possible from histopathology reports, laparoscopy was used as a single reference standard for subgroup analysis. A probability value of < 0.05 was considered statistically significant.

## 3 Results

Of the 2461 participants enrolled in the NECST registry, 436 were eligible for inclusion, with 245 women in the first and 225 in the subsequent laparoscopy groups, respectively. Reasons for exclusion were incomplete initial enrolment, missing demographics, no available ultrasound or laparoscopy report, or not meeting eligibility criteria. The generalised linear mixed model was not significant (*p* = 0.543), demonstrating independence between the two study groups. Women in the subsequent laparoscopy group were older with a higher body mass index (Table 1).

**TABLE 1:** Participant characteristics, and ultrasound and laparoscopy details.

Variable	First laparoscopy (*n* = 245)	Subsequent laparoscopy (*n* = 225)	*p*
**Demographics**			
Age, years ± SD^b^	32.8 ± 7.3	36.0 ± 7.6	< 0.001
BMI, kg/m^2^, median (IQR)^c^	23.9 (21.1–27.5)	25.9 (22.7–30.0)	< 0.001
Smoking status, *n* (%)^a^			0.038
Current smoker	17 (7.0)	22 (10.0)	
Ex‐smoker	44 (18.0)	57 (25.9)	
Non‐smoker	183 (75.0)	141 (64.1)	
Parity, median (IQR)^c^	2 (1–2)	2 (1–2)	0.448
Symptoms, *n* (%)^a^			
Dysmenorrhoea	207 (84.5)	192 (85.3)	0.799
Dyspareunia	163 (66.5)	155 (68.9)	0.585
Dyschezia	156 (63.7)	161 (71.6)	0.068
Dysuria	64 (26.1)	94 (41.8)	< 0.001
Infertility	76 (31.0)	88 (39.1)	0.066
**Ultrasound**			
Endometriosis, *n* (%)^a^	99 (40.4)	101 (44.9)	0.326
Other pathology, *n* (%)^a^			
Leiomyoma	48 (19.6)	42 (18.7)	0.799
Adenomyosis	35 (14.3)	50 (22.2)	0.026
Ovarian cyst	64 (26.1)	47 (20.9)	0.182
Other (e.g., hydrosalpinx, polyp, congenital malformation)	37 (15.1)	39 (17.3)	0.512
None	56 (22.9)	42 (18.7)	0.264
Location of endometriosis, *n* (%)			
Endometrioma^a^	67 (27.3)	64 (28.4)	0.791
USL^a^	36 (14.7)	24 (10.7)	0.191
Bowel^a^	14 (5.7)	27 (12.0)	0.016
Vagina/rectovaginal/retrocervix^a^	12 (4.9)	15 (6.7)	0.411
Bladder^d^	0 (0.0)	4 (1.8)	0.036
Endometriosis markers, *n* (%)			
Kissing ovaries^d^	6 (2.4)	0 (0.0)	0.018
Negative sliding sign^a^	14 (5.7)	20 (8.9)	0.184
Negative anterior sliding sign^a^	7 (2.9)	10 (4.4)	0.357
Tenderness (any location)^a^	54 (22.0)	43 (19.1)	0.433
**Laparoscopy**			
Surgeon, *n* (%)^a^			0.007
Consultant	192 (85.0)	200 (93.0)	
Registrar/fellow	34 (15.0)	15 (7.0)	
Endometriosis, *n* (%)^a^	241 (98.4)	210 (93.3)	0.006
Pathology, *n* (%)^a^			
Leiomyoma	19 (7.8)	8 (3.6)	0.051
Adenomyosis	34 (13.9)	30 (13.3)	0.864
Ovarian cyst	11 (4.5)	7 (3.1)	0.437
Other	9 (3.7)	10 (4.4)	0.672
None	3 (1.2)	13 (5.8)	0.007
Location of endometriosis, *n* (%)			
Pelvic sidewall^a^	164 (66.9)	142 (63.1)	0.384
USL^a^	143 (58.4)	114 (50.7)	0.094
Ovary^a^	116 (47.3)	115 (51.1)	0.415
Fallopian tube^a^	14 (5.7)	16 (7.6)	0.422
Bowel^a^	43 (17.6)	40 (17.8)	0.949
Bladder^a^	14 (5.7)	11 (4.9)	0.690
Ureter^a^	40 (16.3)	50 (22.2)	0.105
Diaphragm^a^	6 (2.4)	9 (4.0)	0.339
Abdominal wall^d^	4 (1.6)	4 (1.8)	1.000
POD^a^	151 (61.6)	130 (57.8)	0.395
Vagina/cervix^a^	11 (4.5)	13 (6.2)	0.403
Other^a^	45 (18.4)	36 (16.0)	0.497
rARSM stage, *n* (%)^a^			0.305
I–II	114 (56.7)	90 (51.4)	
III–IV	87 (43.3)	85 (48.5)	
Phenotype, *n* (%)^a^			0.315
Superficial	65 (32.0)	46 (27.1)	
Deep (excluding endometrioma)	81 (39.9)	81 (47.6)	
Ovarian endometrioma	57 (28.1)	43 (25.3)	
Method of treatment, *n* (%)^a^			0.248
Ablation	11 (5.1)	4 (2.1)	
Excision	121 (56.5)	114 (61.0)	
Combination	82 (38.3)	69 (36.9)	

Abbreviations: BMI, body mass index; IQR, interquartile range; POD, pouch of Douglas; rARSM, revised American Society for Reproductive Medicine; SD, standard deviation; USL, uterosacral ligament.

^a^ Chi‐square test.

^b^ Independent‐samples *t*‐test.

^c^ Mann–Whitney *U* test.

^d^ Fisher's exact test.

Ultrasound was performed a similar number of days prior to laparoscopy in the two groups (first laparoscopy: median 162 days [interquartile range 70–361] vs. subsequent laparoscopy: 160 days [70–367.5]). The incidence of endometriosis as diagnosed by ultrasound did not differ between the two groups. Women in the subsequent laparoscopy group were more likely to be diagnosed with bowel and bladder endometriosis and adenomyosis based on imaging, but these findings did not correlate with intraoperative findings, with similar distributions of locations of endometriosis and other pathology between the two groups at laparoscopy (Table 1). Compared to histopathology findings where concomitant hysterectomy was undertaken, the accuracy of TVUS for detecting adenomyosis was 55.0% (first laparoscopy 53.3% vs. subsequent laparoscopy group 55.6%).

**FIGURE 1:**
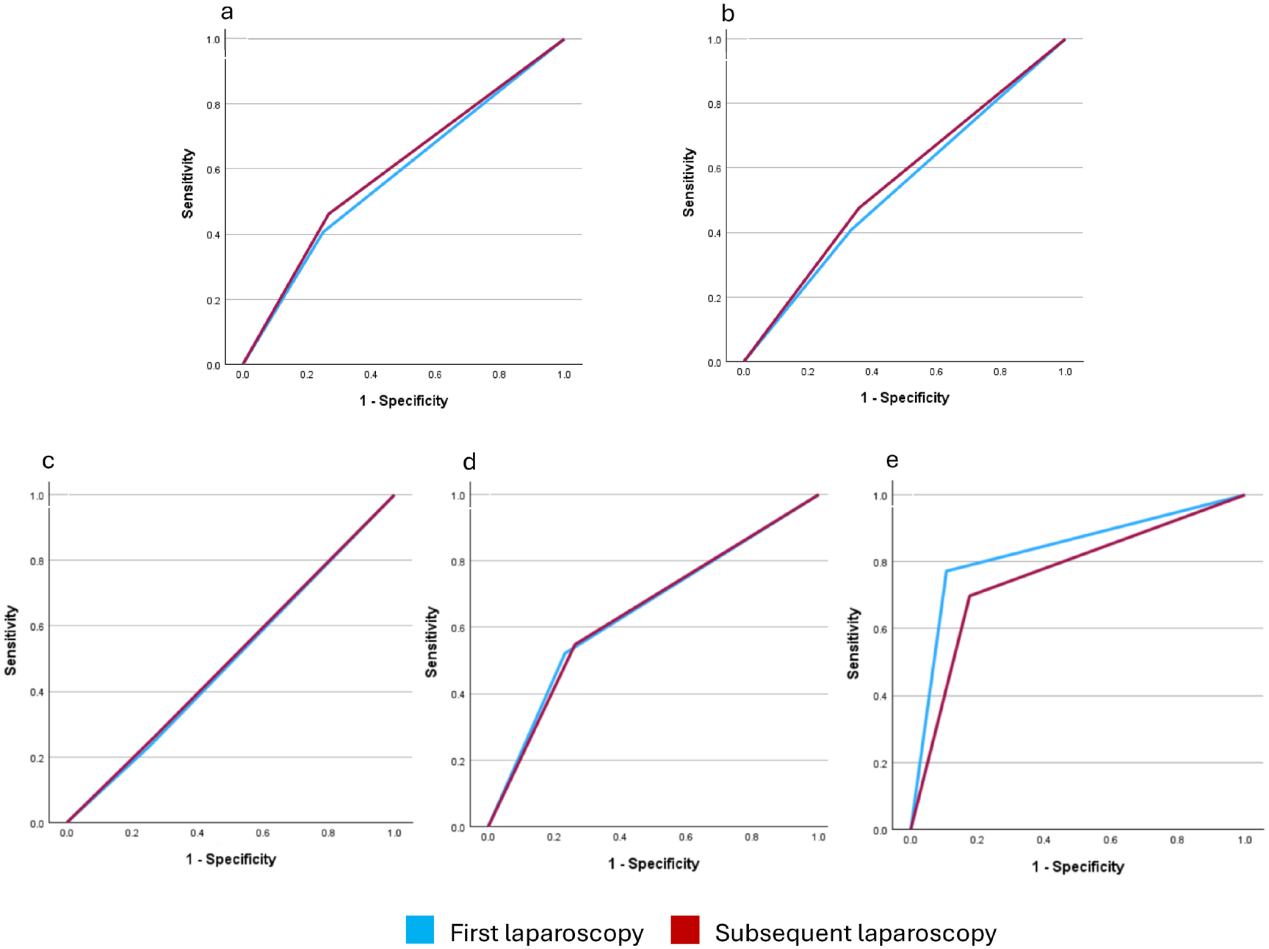
Receiver operating characteristic curve of the diagnostic accuracy of transvaginal ultrasound for endometriosis. (a) Compared to laparoscopy, pelvic endometriosis: First laparoscopy area under the curve 0.58 (95% confidence interval 0.31–0.85) versus subsequent laparoscopy 0.60 (0.46–0.74). (b) Compared to histopathology, pelvic endometriosis: First laparoscopy 0.54 (0.31–0.77) versus subsequent laparoscopy 0.56 (0.45–0.67). (c) Compared to laparoscopy, superficial endometriosis: First laparoscopy 0.49 (0.19–0.79) versus subsequent laparoscopy 0.50 (0.33–0.67). (d) Compared to laparoscopy, deep infiltrating endometriosis: First laparoscopy 0.65 (0.57–0.72) versus subsequent laparoscopy 0.64 (0.56–0.73). (e) Compared to laparoscopy, ovarian endometrioma: First laparoscopy 0.83 (0.76–0.90) versus subsequent laparoscopy 0.76 (0.67–0.85).

Women in the subsequent laparoscopy group were more likely to have a negative laparoscopy, both for endometriosis and any pathology (Table 1). Of those not diagnosed with endometriosis at the time of laparoscopy, in the first laparoscopy group, 2/4 (50.0%) underwent diagnostic laparoscopy, 1/4 (25.0%) cystectomy and 1/4 (25.0%) peritoneal adhesiolysis and tubal dye studies. In the subsequent laparoscopy group, 6/15 (40.0%) underwent hysterectomy, 3/15 (20.0%) peritoneal adhesiolysis, 2/15 (13.3%) bilateral salpingectomy, 1/15 (6.7%) excision of a peritoneal inclusion cyst, 1/15 (6.7%) adenomyomectomy and 2/15 (13.3%) diagnostic laparoscopy. Diagnostic accuracy of TVUS did not differ between the two groups when compared to laparoscopy (Table 2; Figure 1).

**TABLE 2:** Diagnostic accuracy of transvaginal ultrasound for endometriosis.

Reference standard	Laparoscopy		Histopathology	
	First laparoscopy (*n* = 245)	Subsequent laparoscopy (*n* = 225)	First laparoscopy (*n* = 234)	Subsequent laparoscopy (*n* = 209)
Accuracy, % (95% CI)	41.2 (35.1–47.4)	48.0 (41.5–54.5)	41.5 (35.1–47.8)	49.8 (43.0–56.5)
Sensitivity, % (95% CI)	40.7 (34.7–47.0)	46.2 (39.6–52.9)	40.8 (34.6–47.3)	47.5 (40.4–54.8)
Specificity, % (95% CI)	75.0 (29.0–96.0)	73.3 (47.5–89.3)	66.7 (29.6–90.4)	64.3 (45.7–79.3)
PPV (95% CI)	0.99 (0.97–1.00)	0.96 (0.92–1.00)	0.98 (0.95–1.00)	0.90 (0.84–0.96)
NPV (95% CI)	0.02 (0.00–0.04)	0.09 (0.04–0.14)	0.03 (0.00–0.06)	0.16 (0.09–0.23)
LR+ (95% CI)	1.63 (0.30–8.94)	1.73 (0.74–4.06)	1.22 (0.39–3.84)	1.33 (0.79–2.24)
LR− (95% CI)	0.79 (0.45–1.41)	0.73 (0.53–1.02)	0.89 (0.50–1.58)	0.82 (0.60–1.18)

Abbreviations: CI, confidence interval; LR+, positive likelihood ratio; LR−, negative likelihood ratio; NPV, negative predictive value; PPV, positive predictive value.

Histopathology was available for 234/245 (95.5%) women in the first and 209/225 (92.9%) in the subsequent laparoscopy groups. The incidence of endometriosis at histopathology was 92.3% (409/443; first laparoscopy: 97.4% vs. subsequent laparoscopy: 86.6%, *p* < 0.001). Women in the subsequent laparoscopy group were more likely to have fallopian tube endometriosis (3.8% vs. 11.5%, *p* = 0.002). There was no difference in the distribution of ovarian (22.2% vs. 19.1%, *p* = 0.424), bowel (9.8% vs. 11.5%, *p* = 0.572) or rectovaginal septum/vaginal endometriosis (8.5% vs. 5.3%, *p* = 0.176). The diagnostic accuracy of TVUS compared to histopathology did not differ between the two groups (Table 2; Figure 1). Histopathology was not missing at random (*p* < 0.001). There was no difference in diagnostic accuracy of TVUS between the two groups when best‐case/worse‐case scenario analysis was performed (best‐case: first laparoscopy AUC 0.54 [95% CI 0.31–0.77] vs. subsequent laparoscopy 0.55 [0.44–0.67]; worst case: 0.53 [0.39–0.67] vs. 0.57 [0.47–0.66]).

There were 392 cases available for subgroup analysis (first laparoscopy *n* = 207 vs. subsequent laparoscopy *n* = 185). When stratified by phenotype, diagnostic accuracy did not differ between the two groups apart from the NPV for superficial disease, which was higher in the subsequent laparoscopy group. Compared to pelvic endometriosis, accuracy for superficial endometriosis did not differ in any of the diagnostic parameters, while deep and ovarian endometriosis demonstrated improved diagnostics in both groups in some but not all parameters (Table 3; Figure 1).

**TABLE 3:** Diagnostic accuracy of transvaginal ultrasound by phenotype of disease.

Phenotype	Superficial endometriosis		Deep endometriosis		Ovarian endometrioma	
	First laparoscopy (*n* = 69)	Subsequent laparoscopy (*n* = 61)	First laparoscopy (*n* = 207)	Subsequent laparoscopy (*n* = 185)	First laparoscopy (*n* = 207)	Subsequent laparoscopy (*n* = 185)
Accuracy, % (95% CI)	26.1 (15.7–36.4)	37.7 (25.5–49.9)	60.4 (53.7–67.0)	61.1 (54.1–68.1)	86.0 (81.3–90.7)	79.5 (73.6–85.3)
Sensitivity, % (95% CI)	23.1 (14.5–34.8)	26.1 (15.6–40.4)	52.2 (43.9–60.3)	54.8 (46.1–63.3)	77.2 (64.6–86.2)	69.8 (54.8–81.4)
Specificity, % (95% CI)	75.0 (29.0–96.0)	73.3 (47.5–89.3)	76.8 (65.5–85.2)	73.8 (61.4–83.2)	89.3 (83.2–93.4)	82.4 (75.2–87.8)
PPV (95% CI)	0.94 (0.82–1.00)	0.75 (0.54–0.96)	0.82 (0.74–0.90)	0.81 (0.73–0.89)	0.73 (0.62–0.85)	0.55 (0.41–0.68)
NPV (95% CI)	0.06 (0.00–0.12)	0.24 (0.12–0.37)	0.45 (0.36–0.54)	0.45 (0.35–0.54)	0.91 (0.87–0.96)	0.90 (0.85–0.95)
LR+ (95% CI)	0.92 (0.16–5.34)	0.98 (0.37–2.58)	2.25 (1.42–3.56)	2.09 (1.33–3.28)	7.24 (4.46–11.74)	3.96 (2.64–5.95)
LR− (95% CI)	1.03 (0.57–1.83)	1.01 (0.71–1.43)	0.62 (0.50–0.77)	0.61 (0.48–0.78)	0.26 (0.16–0.41)	0.37 (0.23–0.58)

Abbreviations: CI, confidence interval; LR+, positive likelihood ratio; LR−, negative likelihood ratio; NPV, negative predictive value; PPV, positive predictive value.

## 4 Discussion

This study found no difference in the diagnostic accuracy of TVUS for detecting endometriosis in women undergoing a first laparoscopy compared to those with a history of previous surgical treatment. These are the first data to investigate this outcome, with important, clinically relevant findings. TVUS may be used with equal accuracy for people who are surgically naïve compared with those who have previously surgically confirmed endometriosis.

In the 2025 iteration of RANZCOG's Australian endometriosis guidelines [3], TVUS is recommended as a first‐line diagnostic investigation. However, these analyses of national registry data collating community‐based ultrasound found that outside a research setting, diagnostic accuracy is substantively poorer than published data, missing 55.5% of endometriosis. This limitation of the clinical utility of TVUS as a diagnostic test for endometriosis must be acknowledged, given the implications that delayed diagnosis has on the quality of life of patients [12].

In a 2016 Cochrane meta‐analysis of four studies including 512 participants, the sensitivity and specificity of TVUS for diagnosing pelvic endometriosis of any phenotype were 0.79 (95% CI 0.36–1.00) and 0.91 (95% CI 0.74–1.00), respectively [10]. This is considerably higher than the diagnostic accuracy in this study, in which sonographers and sonologists were unaware they were being studied. Our study likely better reflects the ‘real‐world’ accuracy of TVUS that gynaecologists encounter in everyday practice [3, 13, 14]. Given the operator dependency of ultrasound for accurate endometriosis diagnosis, the Hawthorne effect may impact sonographic examinations and account for the considerable difference in this community‐acquired sample compared with optimised imaging studies. Issues of access to the service and the skill set of sonographers and sonologists are factors in diagnostic accuracy, and this may be particularly important in regional and rural Australia.

Laparoscopy and histopathology continue to outperform TVUS, with the important clinical translation for clinicians that a negative ultrasound does not exclude endometriosis. Additionally, there appears to be a potential for false‐positive sonographic diagnosis in subsequent surgery relating to infiltrating disease in bladder or bowel, potentially due to previous fibrotic changes and clinicians should be aware of this when planning surgery. Findings in this study are similar to retrospective data from 294 surgically naïve women with suspected endometriosis and a normal TVUS where laparoscopy confirmed histologically determined endometriosis 52% of the time [15]. Importantly, Australian and international guidelines [3, 13, 14] recommend initiation of first‐line symptom management without initial investigation by laparoscopy, as there is no evidence suggesting superiority of either medical or surgical management [3, 14]. In the case of negative TVUS, given the probability of endometriosis, women should continue to be managed empirically according to Australian guidelines, with laparoscopy considered if symptoms persist despite medical management [3]. Recent mainstream media coverage has raised concerns around diagnosis of endometriosis and interventions, with the Victorian Premier calling for a change to guidelines that mandate ultrasound for diagnosis [16]. Data from this study reinforce that current guidance statements are indeed robust, and it is essential that more scientific study is undertaken for better diagnostic tests for endometriosis. Recent studies report high diagnostic accuracy for a salivary‐based test [17] and blood test [18], with validation studies still required. Magnetic resonance imaging has been shown to be reliably accurate in diagnosing deep endometriosis, but is limited in detecting superficial endometriosis, with the additional barrier of inequitable access [3, 19].

This study did not find a difference in diagnostic accuracy between groups for any phenotype, recognising that power is a limiting factor for subgroup analyses. From a meta‐analysis of 21 prospective studies including 3847 participants, the sensitivity of detecting deep endometriosis with TVUS was 0.76 (95% CI 0.67–0.83), with a specificity of 0.94 (0.88–0.97) [19]. In a 2016 Cochrane meta‐analysis of eight studies including 765 participants, ovarian disease was detected with a sensitivity of 0.93 (95% CI 0.87–0.99) and a specificity of 0.96 (0.92–0.99) [10]. Studies exploring the diagnostic accuracy of TVUS for superficial endometriosis are few. A retrospective study of 100 women reports a sensitivity of 0.52 (95% CI 0.34–0.69), and a specificity of 0.94 (0.85–0.98) [20]. When assessed by phenotype from the presented study, TVUS failed to identify 63.2% of laparoscopically confirmed superficial endometriosis, which is estimated to be the single disease subtype in 31% of women in both published [21] and this (29.8%) study. Even when considering deep endometriosis, this real‐world study of sonography misdiagnosed 39.2% of disease, highlighting the disconnect between best‐case scenarios in research studies and clinical practice that patients rely on. It is unclear whether the introduction of the new Medicare Benefits Schedule item for ultrasound assessment of complex gynaecological conditions introduced on 1 November 2025 [22] will affect these data, and it is apparent that considerable education is required to bridge the gap between the optimal outcome and current practice in Australia.

The strengths of this study include its large size, multicentre recruitment, representative mix of disease severity, variable skill levels of sonologists, sonographers and surgeons and use of two reference standards. The study limitations include a lack of blinding to previous ultrasound or surgical diagnoses, although doing so would be neither practical nor ethical as both are paramount to surgical planning and patient safety, regardless of study design. The retrospective nature of the study has limitations; however, the binary outcome of surgically and histologically confirmed disease reduces this impact, while capturing community‐acquired data. The 161‐day delay between TVUS and laparoscopy noted in the study could be an issue if the course of disease progression were linear. However, pooled outcomes from seven randomised trials in women undergoing laparoscopy where no treatment was performed (medical or surgical) for 6 months showed no progression in 71% of participants [23]. These data indicate that the delay from ultrasound to surgery in this study is unlikely to substantially contribute to the poorer diagnostic outcomes observed.

In summary, this study found no difference in the diagnostic accuracy of TVUS for detecting endometriosis in women undergoing a first laparoscopy compared to those with a history of previous surgical treatment. However, based on nationally sourced registry data in Australia, community ultrasound appears to have significant limitations for diagnostic purposes, even for deep endometriosis and particularly for superficial disease. These data reinforce recommendations from RANZCOG's Australian endometriosis guidelines [3] that a negative ultrasound does not exclude endometriosis, and patients should continue to be treated under a presumptive diagnosis. This must be considered by clinicians when counselling women presenting with suspected or recurrent endometriosis.

## Funding

This research was funded in part by the Medical Research Future Fund (MRFAR000210) and the Australian Government Department of Health, Disability and Ageing's Public Health and Chronic Disease Program (4‐I66SNMA).

## Conflicts of Interest

Jason A. Abbott is on advisory boards for Gedeon Richter, BD and Hologic. He is on the Endometriosis Advisory Group for the Australian Government, chaired the first Australian Guidelines for Endometriosis Diagnosis and Management and contributed to the Government's National Action Plan on Endometriosis. He is the chair of the NECST Network and holds multiple competitive grants in endometriosis research through Australian Government funding agencies. Cecilia Ng manages research grant funding from the MRFF and was a previous employee with CSL Vifor (formerly Vifor Pharma Pty. Ltd.).

## Data Availability

The data that support the findings of this study are available from the corresponding author upon reasonable request.
